# Zn_1.86_Cd_0.14_(OH)VO_4_

**DOI:** 10.1107/S1600536810044806

**Published:** 2010-11-10

**Authors:** Tamara Đorđević, Jovica Stojanović, Ljiljana Karanović

**Affiliations:** a Institut für Mineralogie und Kristallographie, Universität Wien-Geozentrum, Althanstrasse 14, A-1090 Vienna, Austria; b Applied Mineralogy Unit, Institute for Technology of Nuclear and Other Mineral Raw Materials, Franchet ď Eperey 86, PO Box 390, 11000 Belgrade, Serbia; c Laboratory of Crystallography, Faculty of Mining and Geology, Đušina 7, 11000 Belgrade, Serbia

## Abstract

The title compound, dizinc cadmium hydroxide tetraoxido­vanadate, Zn_1.86_Cd_0.14_(OH)VO_4_, was prepared under low-temperature hydro­thermal conditions. It is isostructural with Zn_2_(OH)VO_4_ and Cu_2_(OH)VO_4_. In the crystal structure, chains of edge-sharing [ZnO_6_] octahedra are inter­connected by VO_4_ tetra­hedra (site symmetries of both V atoms and their coordination polyhedra are .*m*.) to form a three-dimensional [Zn(OH)VO_4_]^2−^ framework with channels occupied by Zn and Zn/Cd cations adopting trigonal–bipyramidal and distorted octa­hedral coordinations, respectively. Zn_1.86_Cd_0.14_(OH)VO_4_ is topologically related to adamite-type phases, and descloizite- and tsumcorite-type structures.

## Related literature

For isostructural compounds, see: Wang *et al.* (1998 ▶); Wu *et al.* (2003 ▶). For topologically related structures, see: Nandini & Vidyasagar (1998 ▶); Bachmann (1953 ▶); Qurashi & Barnes (1964 ▶). For structurally related compounds, see: Hawthorne & Faggiani (1979 ▶); Tillmanns & Gebert (1973 ▶). For bond-valence analysis, see: Brese & O’Keeffe (1991 ▶). 

## Experimental

### 

#### Crystal data

Zn_1.86_Cd_0.14_(OH)VO_4_
*M* *_r_* = 535.62
Orthorhombic, 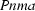

*a* = 14.702 (3) Å
*b* = 6.0511 (12) Å
*c* = 8.9460 (18) Å
*V* = 795.8 (3) Å^3^
*Z* = 4
Mo *K*α radiation
μ = 14.00 mm^−1^
*T* = 293 K
0.18 × 0.03 × 0.02 mm

#### Data collection

Nonius KappaCCD diffractometer
Absorption correction: multi-scan (Otwinowski & Minor, 1997 ▶; Otwinowski *et al.*, 2003 ▶) *T*_min_ = 0.187, *T*_max_ = 0.767
5550 measured reflections
1566 independent reflections
1377 reflections with *I* > 2σ(*I*)
*R*_int_ = 0.013

#### Refinement

*R*[*F* ^2^ > 2σ(*F* ^2^)] = 0.025
*wR*(*F* ^2^) = 0.056
*S* = 1.17
1566 reflections
90 parameters
2 restraints
H-atom parameters constrained
Δρ_max_ = 0.79 e Å^−3^
Δρ_min_ = −0.91 e Å^−3^

### 

Data collection: *COLLECT* (Nonius, 2002 ▶); cell refinement: *SCALEPACK* (Otwinowski & Minor, 1997 ▶); data reduction: *DENZO-SMN* (Otwinowski & Minor, 1997 ▶); program(s) used to solve structure: *SIR97* (Altomare *et al.*, 1999 ▶); program(s) used to refine structure: *SHELXL97* (Sheldrick, 2008 ▶) and *WinGX* (Farrugia, 1999 ▶); molecular graphics: *ATOMS* (Dowty, 2000 ▶); software used to prepare material for publication: *publCIF* (Westrip, 2010 ▶).

## Supplementary Material

Crystal structure: contains datablocks global, I. DOI: 10.1107/S1600536810044806/br2149sup1.cif

Structure factors: contains datablocks I. DOI: 10.1107/S1600536810044806/br2149Isup2.hkl

Additional supplementary materials:  crystallographic information; 3D view; checkCIF report

## Figures and Tables

**Table 1:** Hydrogen-bond geometry (Å, °)

*D*—H⋯*A*	*D*—H	H⋯*A*	*D*⋯*A*	*D*—H⋯*A*
O7—H1⋯O4^i^	0.89 (2)	2.45 (2)	3.176 (3)	139 (1)
O7—H1⋯O4^ii^	0.89 (2)	2.45 (2)	3.176 (3)	139 (1)
O8—H2⋯O2	0.88 (2)	1.84 (2)	2.708 (4)	175 (9)

Symmetry codes: (i) 
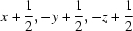
; (ii) 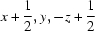.

**Fig. 1.**
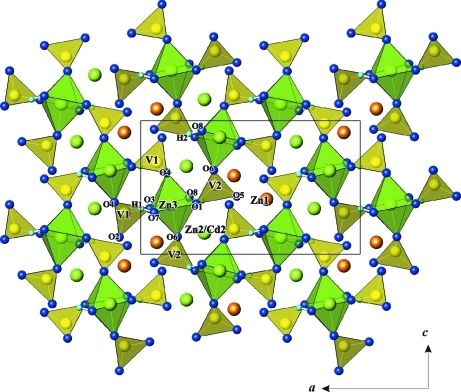
Polyhedral view of the structure of Zn1.86Cd0.14(OH)VO4 along [010].

**Fig. 2.**
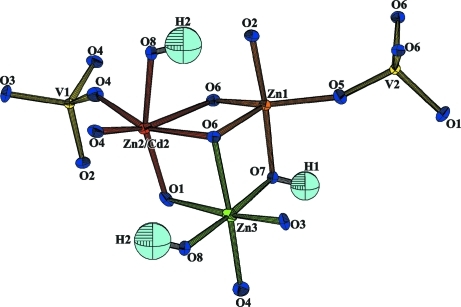
The local coordination of V, Zn and Cd atoms with atomic displacement ellipsoids at 50% probability.

## supplementary crystallographic information

### Comment

The phases in *A*–*M*–*X*–O–(H) system often form such family of compounds showing rich structural chemistry with anionic frameworks built from *M*O_6_ octahedra and *X*O_4_ tetrahedra and *A*^n+^ ions as counter cations. There are many reports on divalent metal vanadates synthesized by high temperature solid state reactions. However, hydrothermal methods are proved to be effective for the synthesis of new vanadium compounds, including zinc vanadates (Wang *et al.*, 1998 and references therein). To keep the products of hydrothermal synthesis under control is often difficult because of the high sensitivity to the exact reaction conditions. However, hydrothermal syntheses often result in well developed single crystals. Here we report on the new zinc cadmium hydrogen vanadate, (Zn_1.86_Cd_0.14_)(OH)VO_4_. In its crystal structure [Zn3O_6_]*_n_* octahedral chains are interconnected by VO_4_ tetrahedra to form a [Zn3(OH)VO_4_] framework. The voids are filled by Zn1 and Zn2/Cd2 cations with trigonal bipyramidal and distorted octahedral coordination, respectively. The two distinct V atoms adopt tetrahedral coordination. VO_4_ tetrahedra are distorted and both have site symmetry .*m*. V—O bond lengths are in the ranges of 1.684 (3) to 1.729 (2) Å for V1 and 1.651 (3) to 1.789 (3) Å for V2. The Zn—O bond lengths vary from 1.958 (3) to 2.427 (2) Å. (Zn_1.86_Cd_0.14_)(OH)VO_4_ is isostructural with Zn_2_(OH)VO_4_ (Wang *et al.*, 1998) and Cu_2_(OH)VO_4_ (Wu *et al.*, 2003) and topologically related to *A*SbV_2_O_8_ (*A* = K, Rb, Tl or Cs) (Nandini & Vidyasagar, 1998), adamite-type phases (Zn_2_(*X*O_4_)(OH), *X*^5+^ = P, As, V) and the minerals descloizite PbZn(VO_4_)(OH) (Bachmann, 1953; Qurashi & Barnes, 1964; Hawthorne & Faggiani, 1979) and tsumcorite PbZn_2_(AsO_4_)_2_(H_2_O) (Tillmanns & Gebert, 1973). In descloizite- and adamite-type structures the [ZnV_2_O_9_]-type chain is linked to four neighbours by sharing one column of tetrahedra with each neighbour. In the title compound the [Zn3V_2_O_9_] chain is linked to three neighbours by sharing two columns tetrahedra with one neighbour and one column with each of the other two neighbours (see Figs. 4 and 5 in Wang *et al.*, 1998). If [ZnV_2_O_9_]-type chain shares two columns of tetrahedra with all neighbours, a two-dimensional layer instead of three-dimensional framework are formed. Such case is found in mineral tsumcorite, where [ZnAs_2_O_9_] chain is linked by sharing two of AsO_4_ tetrahedra with each of its two neighbours thus forming a layered structure eighbor and one column with each of the other two neighbours (see Fig. 6 in Wang *et al.*, 1998). Bond-valence summations for all atoms, calculated using the parameters of Brese & O'Keeffe (1991), give 2.00 v.u. (valence units) for Zn1, 2.00 (1.22/0.78) v.u.for Zn2/Cd2, 2.07 v.u. for Zn3, 5.11 v.u. for V1, 4.90 for V2. For O atoms bond-valence summations are 1.94 v.u. (O1), 1.88 v.u. (O2), 1.99 v.u. (O3), 1.94 v.u. (O4), 1.96 v.u. (O5), 1.90 (O6), 1.32 v.u. (O7) and 1.38 v.u. (O8). Taking into account that the O7 and O8 atoms are the single donors of strong hydrogen bonds toward O4 (H2 forms a bifurcated hydrogen bond to two O4 atoms) and O2, respectively, the bond valences are well balanced.

### Experimental

Single crystals of (Zn_1.86_Cd_0.14_)(OH)VO_4_ were obtained as reaction products from mixtures of Cd(OH)_2_ (Alfa Products), 2ZnO.2CO_3_.4H_2_O (Alfa Products), and V_2_O_5_ (Fluka Chemika 94710, 98%). The mixture was transferred into Teflon vessel and filled to approximately 70% of their inner volume with distilled water (pH of the mixture was 6). Finally it was enclosed into stainless steel autoclave. The mixture was heated under heating regime with three steps: the autoclaves were heated from 293.15 to 473.15 K (4 h), held at 473.15 K  for 192 h, and finally cooled to room temperature within 175 h. At the end of the reaction the pH of the solvent was 6. The reaction products were filtered and washed thoroughly with distilled water. (Zn_1.86_Cd_0.14_)(OH)VO_4_ crystallized as transparent colourless needle-like crystals (yield *ca* 65%) and uninvestigated powder (yield *ca* 35%). All crystals are up to 0.2 mm in length.

Qualitative chemical analyses were performed using a Jeol JSM-6400LV scanning electron microscope (SEM) connected with a LINK energy-dispersive X-ray analysis (EDX) unit confirmed the presence of Zn, Cd and V.

### Refinement

Studies of several single crystals of (Zn_1.86_Cd_0.14_)(OH)VO_4_ all revealed orthorhombic unit cell. A sample exhibiting sharp reflection spots was chosen for data collection. The crystal structure was refined starting from the atomic coordinates of Zn_2_(OH)VO_4_ (Wang *et al.*, 1998) using standard procedures. The space-group symmetry *Pnma* was indicated by systematic absences and intensity statistics, and was confirmed by the structure refinement. Substitutional disorder was apparent and the occupancies of Zn2^2+^ and Cd2^2+^ were refined keeping the occupancy sum of Zn2+Cd2 fixed at 2.0 atoms per unit cell to satisfy the charge balance. The atomic coordinates and displacement parameters of Zn2 and Cd2 were kept equal. Occupancy of 72.7 and 27.3% for Zn2 and Cd2, respectively, were obtained. Anisotropic displacement parameters were allowed to vary for all non-H atoms. The H atoms were located from difference Fourier map and refined as riding atoms, with restraints on the O—H bond distance of 0.82 (2) Å and *U*_iso_(H) values at 1.2*U*_eq_(O).

### Crystal data

**Table:**

Zn_1.86_Cd_0.14_(OH)VO_4_	*F*(000) = 1010
*M**_r_* = 535.62	*D*_x_ = 4.470 Mg m^−^^3^
Orthorhombic, *P**n**m**a*	Mo *K*α radiation, λ = 0.71073 Å
Hall symbol: -P 2ac 2n	Cell parameters from 1706 reflections
*a* = 14.702 (3) Å	θ = 0.4–32.6°
*b* = 6.0511 (12) Å	µ = 14.00 mm^−^^1^
*c* = 8.9460 (18) Å	*T* = 293 K
*V* = 795.8 (3) Å^3^	Prismatic, colourless
*Z* = 4	0.18 × 0.03 × 0.02 mm

### Data collection

**Table:**

Nonius KappaCCD diffractometer	1566 independent reflections
Radiation source: fine-focus sealed tube	1377 reflections with *I* > 2σ(*I*)
graphite	*R*_int_ = 0.013
φ and ω scans	θ_max_ = 32.6°, θ_min_ = 2.8°
Absorption correction: multi-scan (Otwinowski & Minor, 1997; Otwinowski *et al.*, 2003)	*h* = −22→22
*T*_min_ = 0.187, *T*_max_ = 0.767	*k* = −9→9
5550 measured reflections	*l* = −13→13

### Refinement

**Table:**

Refinement on *F*^2^	Secondary atom site location: difference Fourier map
Least-squares matrix: full	Hydrogen site location: difference Fourier map
*R*[*F*^2^ > 2σ(*F*^2^)] = 0.025	H-atom parameters constrained
*wR*(*F*^2^) = 0.056	*w* = 1/[σ^2^(*F*_o_^2^) + (0.0223*P*)^2^ + 1.9948*P*] where *P* = (*F*_o_^2^ + 2*F*_c_^2^)/3
*S* = 1.17	(Δ/σ)_max_ < 0.001
1566 reflections	Δρ_max_ = 0.79 e Å^−^^3^
90 parameters	Δρ_min_ = −0.91 e Å^−^^3^
2 restraints	Extinction correction: *SHELXL97* (Sheldrick, 2008), Fc^*^=kFc[1+0.001xFc^2^λ^3^/sin(2θ)]^-1/4^
Primary atom site location: structure-invariant direct methods	Extinction coefficient: 0.00065 (16)

### Special details

**Table:**

Geometry. All esds (except the esd in the dihedral angle between two l.s. planes) are estimated using the full covariance matrix. The cell esds are taken into account individually in the estimation of esds in distances, angles and torsion angles; correlations between esds in cell parameters are only used when they are defined by crystal symmetry. An approximate (isotropic) treatment of cell esds is used for estimating esds involving l.s. planes.
Refinement. Refinement of F^2^ against ALL reflections. The weighted R-factor wR and goodness of fit S are based on F^2^, conventional R-factors R are based on F, with F set to zero for negative F^2^. The threshold expression of F^2^ > 2sigma(F^2^) is used only for calculating R-factors(gt) etc. and is not relevant to the choice of reflections for refinement. R-factors based on F^2^ are statistically about twice as large as those based on F, and R- factors based on ALL data will be even larger.

### Fractional atomic coordinates and isotropic or equivalent isotropic displacement parameters (Å^2^)

**Table:**

	*x*	*y*	*z*	*U*_iso_*/*U*_eq_	Occ. (<1)
Zn1	0.42606 (3)	0.2500	0.41189 (5)	0.01136 (10)	
Zn2	0.20888 (3)	0.2500	0.34164 (4)	0.01276 (13)	0.726 (5)
Cd2	0.20888 (3)	0.2500	0.34164 (4)	0.01276 (13)	0.274 (5)
Zn3	0.36089 (2)	−0.00355 (5)	0.12498 (3)	0.01342 (9)	
V1	0.42663 (4)	0.2500	0.81152 (7)	0.00849 (12)	
V2	0.16102 (4)	0.2500	−0.02047 (7)	0.00833 (12)	
O1	0.24703 (19)	0.2500	0.1209 (3)	0.0133 (5)	
O2	0.4029 (2)	0.2500	0.6273 (3)	0.0158 (5)	
O3	0.45895 (18)	−0.2500	0.1564 (3)	0.0139 (5)	
O4	0.11984 (14)	−0.0146 (3)	0.3903 (2)	0.0162 (4)	
O5	0.56142 (19)	0.2500	0.4353 (3)	0.0172 (6)	
O6	0.33279 (13)	−0.0120 (3)	0.37011 (19)	0.0122 (3)	
O7	0.43897 (17)	0.2500	0.1853 (3)	0.0101 (5)	
H1	0.4931	0.2500	0.1629	0.012*	
O8	0.22122 (18)	0.2500	0.5759 (3)	0.0114 (5)	
H2	0.2752	0.2500	0.5989	0.014*	

### Atomic displacement parameters (Å^2^)

**Table:**

	*U*^11^	*U*^22^	*U*^33^	*U*^12^	*U*^13^	*U*^23^
Zn1	0.01098 (19)	0.0146 (2)	0.00853 (19)	0.000	−0.00083 (14)	0.000
Zn2	0.0144 (2)	0.0143 (2)	0.00956 (19)	0.000	0.00227 (13)	0.000
Cd2	0.0144 (2)	0.0143 (2)	0.00956 (19)	0.000	0.00227 (13)	0.000
Zn3	0.01645 (15)	0.00951 (14)	0.01431 (16)	−0.00237 (11)	−0.00186 (10)	−0.00122 (10)
V1	0.0081 (3)	0.0099 (3)	0.0074 (2)	0.000	0.00004 (19)	0.000
V2	0.0081 (2)	0.0086 (2)	0.0084 (3)	0.000	−0.00083 (19)	0.000
O1	0.0140 (12)	0.0087 (11)	0.0172 (13)	0.000	−0.0071 (10)	0.000
O2	0.0162 (13)	0.0224 (14)	0.0088 (12)	0.000	−0.0011 (10)	0.000
O3	0.0082 (11)	0.0116 (11)	0.0220 (13)	0.000	−0.0041 (10)	0.000
O4	0.0202 (9)	0.0152 (9)	0.0131 (9)	−0.0040 (8)	−0.0016 (7)	−0.0030 (7)
O5	0.0124 (12)	0.0234 (14)	0.0158 (13)	0.000	−0.0033 (10)	0.000
O6	0.0146 (8)	0.0116 (8)	0.0102 (8)	−0.0010 (7)	0.0000 (6)	−0.0006 (6)
O7	0.0085 (11)	0.0098 (11)	0.0118 (11)	0.000	0.0005 (9)	0.000
O8	0.0092 (11)	0.0088 (11)	0.0160 (12)	0.000	−0.0008 (9)	0.000

### Geometric parameters (Å, °)

**Table:**

Zn1—O2	1.957 (3)	Zn3—O4^ii^	2.1214 (19)
Zn1—O5	2.001 (3)	Zn3—O6	2.2321 (18)
Zn1—O7	2.036 (3)	Zn3—O1	2.271 (2)
Zn1—O6	2.1291 (19)	V1—O2	1.685 (3)
Zn1—O6^i^	2.1291 (19)	V1—O3^iii^	1.706 (3)
Zn2—O1	2.053 (3)	V1—O4^iv^	1.7299 (19)
Zn2—O4	2.113 (2)	V1—O4^v^	1.730 (2)
Zn2—O4^i^	2.113 (2)	V2—O5^vi^	1.650 (3)
Zn2—O6^i^	2.428 (2)	V2—O6^ii^	1.7439 (19)
Zn2—O6	2.428 (2)	V2—O6^vii^	1.7439 (19)
Zn3—O8^ii^	1.9683 (17)	V2—O1	1.788 (3)
Zn3—O3	2.0931 (19)		
			
O2—Zn1—O5	94.01 (12)	O8^ii^—Zn3—O4^ii^	84.27 (9)
O2—Zn1—O7	175.32 (12)	O3—Zn3—O4^ii^	94.46 (10)
O5—Zn1—O7	90.67 (11)	O8^ii^—Zn3—O6	95.08 (9)
O2—Zn1—O6	93.48 (8)	O3—Zn3—O6	88.82 (10)
O5—Zn1—O6	131.22 (5)	O4^ii^—Zn3—O6	176.58 (8)
O7—Zn1—O6	83.41 (7)	O8^ii^—Zn3—O1	93.22 (8)
O2—Zn1—O6^i^	93.48 (8)	O3—Zn3—O1	172.38 (10)
O5—Zn1—O6^i^	131.22 (5)	O4^ii^—Zn3—O1	92.72 (9)
O7—Zn1—O6^i^	83.41 (7)	O6—Zn3—O1	83.96 (9)
O6—Zn1—O6^i^	96.24 (10)	O2—V1—O3^iii^	111.65 (15)
O1—Zn2—O4	111.55 (7)	O2—V1—O4^iv^	108.46 (8)
O1—Zn2—O4^i^	111.55 (7)	O3^iii^—V1—O4^iv^	108.71 (9)
O4—Zn2—O4^i^	98.51 (11)	O2—V1—O4^v^	108.46 (8)
O1—Zn2—O6^i^	84.03 (7)	O3^iii^—V1—O4^v^	108.71 (9)
O4—Zn2—O6^i^	159.66 (7)	O4^iv^—V1—O4^v^	110.86 (14)
O4^i^—Zn2—O6^i^	87.05 (7)	O5^vi^—V2—O6^ii^	107.78 (8)
O1—Zn2—O6	84.03 (7)	O5^vi^—V2—O6^vii^	107.78 (8)
O4—Zn2—O6	87.05 (7)	O6^ii^—V2—O6^vii^	111.37 (12)
O4^i^—Zn2—O6	159.66 (7)	O5^vi^—V2—O1	107.51 (14)
O6^i^—Zn2—O6	81.51 (9)	O6^ii^—V2—O1	111.10 (8)
O8^ii^—Zn3—O3	84.98 (8)	O6^vii^—V2—O1	111.10 (8)

Symmetry codes: (i) *x*, −*y*+1/2, *z*; (ii) −*x*+1/2, −*y*, *z*−1/2; (iii) −*x*+1, −*y*, −*z*+1; (iv) −*x*+1/2, *y*+1/2, *z*+1/2; (v) −*x*+1/2, −*y*, *z*+1/2; (vi) *x*−1/2, *y*, −*z*+1/2; (vii) −*x*+1/2, *y*+1/2, *z*−1/2.

### Hydrogen-bond geometry (Å, °)

**Table:**

*D*—H···*A*	*D*—H	H···*A*	*D*···*A*	*D*—H···*A*
O7—H1···O4^viii^	0.89 (2)	2.45 (2)	3.176 (3)	139 (1)
O7—H1···O4^ix^	0.89 (2)	2.45 (2)	3.176 (3)	139 (1)
O8—H2···O2	0.88 (2)	1.84 (2)	2.708 (4)	175 (9)

Symmetry codes: (viii) *x*+1/2, −*y*+1/2, −*z*+1/2; (ix) *x*+1/2, *y*, −*z*+1/2.
